# Synthesis and *In Vitro* Evaluation of a HER2-Specific ImmunoSCIFI Probe

**DOI:** 10.1021/acsomega.3c06452

**Published:** 2023-12-07

**Authors:** Katie Gristwood, Saimir Luli, Kenneth S. Rankin, James C. Knight

**Affiliations:** † School of Natural and Environmental Sciences, Newcastle University, Bedson Building, Newcastle upon Tyne NE1 7RU, U.K.; ‡ Preclinical In Vivo Imaging, Translational and Clinical Research Institute, Newcastle University, Newcastle upon Tyne NE2 4HH, U.K.; § Translational and Clinical Research Institute, Newcastle University, Newcastle upon Tyne NE1 7RU, U.K.

## Abstract

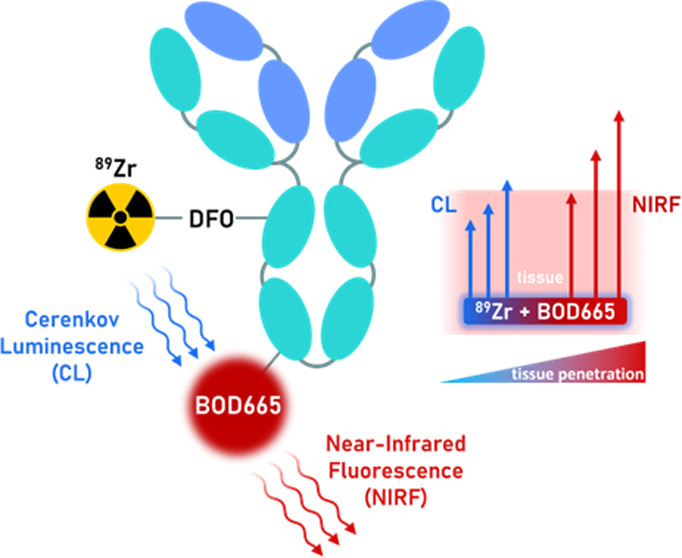

Secondary Cerenkov-induced fluorescence imaging (SCIFI) is an emerging biomedical optical imaging modality that leverages Cerenkov luminescence, primarily generated by β-emitting radioisotopes, to excite fluorophores that offer near-infrared emissions with optimal tissue penetrance. Dual-functionalized immunoconjugates composed of an antibody, a near-infrared fluorophore, and a β-emitting radioisotope have potential utility as novel SCIFI constructs with high specificity for molecular biomarkers of disease. Here, we report the synthesis and characterization of [^89^Zr]Zr-DFO-trastuzumab-BOD665, a self-excitatory HER2-specific “immunoSCIFI” probe capable of yielding near-infrared fluorescence *in situ* without external excitation. The penetration depth of the SCIFI signal was measured in hemoglobin-infused optical tissue phantoms that indicated a 2.05-fold increase compared to ^89^Zr-generated Cerenkov luminescence. Additionally, the binding specificity of the immunoSCIFI probe for HER2 was evaluated in a cellular assay that showed significantly higher binding to SKBR3 (high HER2 expression) relative to MDA-MB-468 (low HER2) breast cancer cells based on measurements of total flux in the near-infrared region with external excitation blocked. Taken together, the results of this study indicate the potential utility of immunoSCIFI constructs for interrogation of molecular biomarkers of disease.

## Introduction

Secondary Cerenkov-induced fluorescence imaging (SCIFI) is a nascent biomedical optical imaging modality that utilizes Cerenkov luminescence (CL) (a low-intensity, blue-weighted light, generated during radioactive decay) to excite clinically relevant dyes with near-infrared (NIR) emissions. Compared to Cerenkov luminescence imaging (CLI), SCIFI offers deep tissue imaging due to the improved penetrability of NIR light through biological tissues, particularly at 650–1350 nm.^1^ While CL-induced fluorescence is low intensity relative to that generated by conventional external excitation sources, SCIFI is less impeded by tissue autofluorescence and reflection artifacts, leading to enhanced signal-to-background ratios.^2^

Seminal work by Liu et al. described efficient CL-mediated excitation of QD655 quantum dots upon subcutaneous and intramuscular coadministration with [^131^I]NaI in athymic nude mice.^3^ Dothager et al. similarly observed red-shifted photonic emissions upon excitation of Qtracker705 by ^18^F- and ^64^Cu-generated CL *in vitro* and in athymic nude mice bearing subcutaneous QD705-containing pseudotumors after administration of [^18^F]FDG.^4^ A study by Thorek et al. reported a SCIFI signal upon colocalization of QD605 (coupled to α_V_β_3_-targeting cyclic-RGD peptide) and [^89^Zr]Zr-DFO-trastuzumab in mice bearing HER2/neu-positive xenograft tumors, in addition to an activatable SCIFI strategy enabling quantification of MMP-2 activity.^2^ Recently, we examined the suitability of boron-dipyrromethene (BODIPY) fluorophores in SCIFI applications based on a panel of *meso*-substituted BODIPY analogues and observed efficient CL-induced fluorescence excitation upon combination with the positron emitting radiometal ^89^Zr.^5^ The BODIPY dyes exhibited high photostability despite prolonged exposure to ionizing radiation. However, these dyes had limited clinical value due to their sub-NIR fluorescence and lack of biomarker specificity.

In the era of precision medicine, antibodies have been widely used as targeting vectors in diagnostic and therapeutic applications due to their ability to bind molecular biomarkers of disease with high specificity. This is exemplified in a related investigation by Meimetis et al., who developed a dual-modal (PET/fluorescence) imaging agent, [^89^Zr]Zr-DFO-trastuzumab-BODIPY, capable of discriminating HER2 expression in breast cancer xenografts in mice. While the BODIPY fluorophore had limited *in vivo* utility due to its sub-NIR emission profile, it was used in *ex vivo* fluorescence analysis of tumor tissue to evaluate tracer distribution.^6^ Similarly, Lee et al. developed a radioimmunoconjugate based on another HER2-specific antibody, pertuzumab, configured for both PET/CLI and NIR imaging via modification with ^89^Zr and IRDye800CW, respectively. Notably, this study included CLI-guided tumor excision that resulted in negative margins.^7^ In contrast, antibodies remain largely unexplored in SCIFI applications, representing a significant gap in the advancement of this emerging optical imaging modality.

We propose that the construction of immunoSCIFI probes can be achieved by conjugation of three requisite components: (i) an antibody or antibody-based species (e.g. antibody fragment), (ii) a CL-generating radioisotope, and (iii) a fluorescent agent compatible with CL excitation.

**Figure 1:**
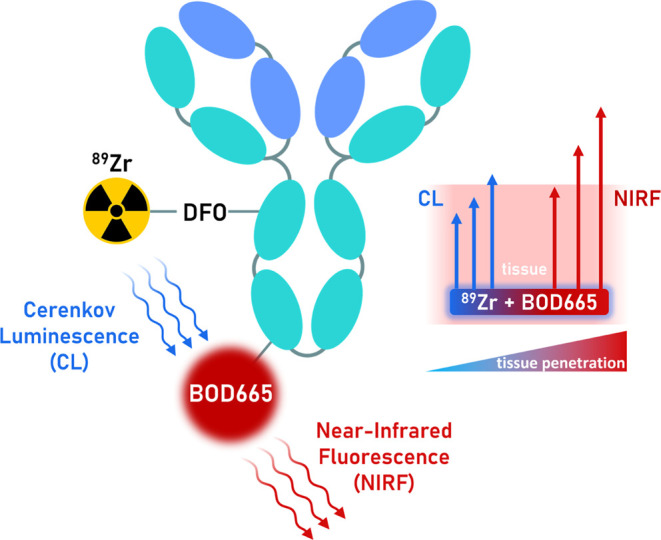
ImmunoSCIFI conjugate showing the energy transfer from ^89^Zr-generated Cerenkov luminescence (CL) to BOD665, yielding near-infrared fluorescence (NIRF) that is able to penetrate further through tissue than CL.

Here, we describe the synthesis and characterization of a novel HER2-targeting immunoSCIFI probe, [^89^Zr]Zr-DFO-trastuzumab-BOD665. This dual-functionalized immunoconjugate combines trastuzumab for HER2 specificity, the β^+^-emitting radioisotope ^89^Zr for generation of Cerenkov luminescence, and the near-infrared fluorophore BOD665 for fluorescence imaging (Figure 1). This investigation includes measurements of the penetration depth of the SCIFI signal using hemoglobin-infused optical tissue phantoms and also evaluates the HER2 specificity of the immunoSCIFI probe via a cell uptake assay.

## Results and Discussion

### Spectroscopic Analysis of BOD665

**Figure 2:**
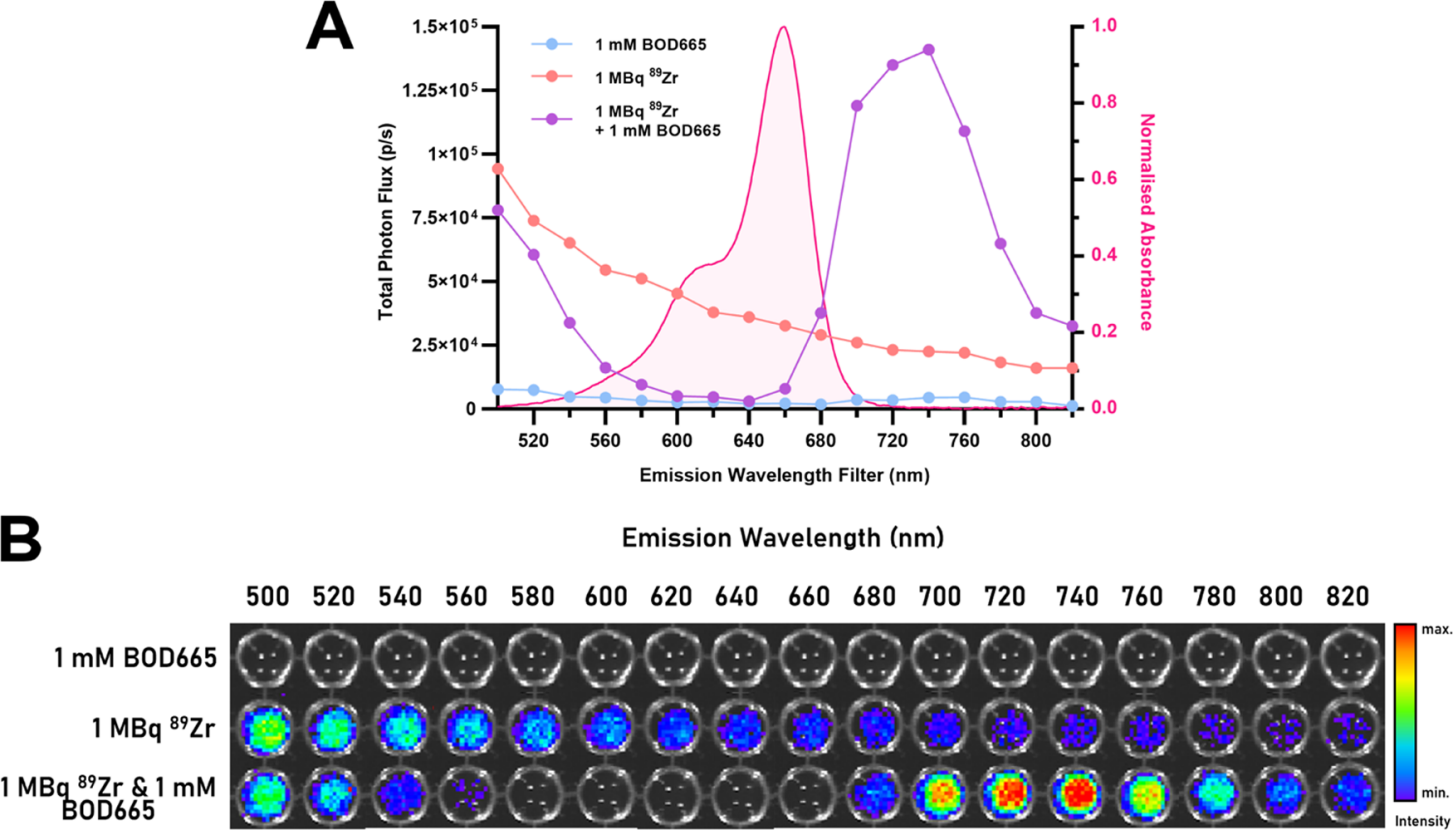
(A) Total photon flux values acquired on an IVIS scanner with emission wavelength filters ranging between 500 to 820 nm (20 nm bandwidth) from solutions of BOD665 (1 mM, blue), ^89^Zr (1 MBq, orange), and BOD665 (1 mM) combined with ^89^Zr (1 MBq) (purple). The absorbance of BOD665 is shown (pink). A reduction in photon flux between 520 and 680 nm observed in the combined solution of BOD665 and ^89^Zr is due to the transfer of Cerenkov luminescence photon energy to the fluorophore. (B) IVIS images of the respective solutions. SCIFI signal in the combined solution of BOD665 and ^89^Zr can be observed at 680–820 nm.

BODIPY 650/665-X NHS ester (BOD665) has two absorption bands that peak at 363 and 655 nm, the former coinciding with blue-weighted (<500 nm) Cerenkov luminescence.^2,8^ The emission maximum of the dye is 697 nm, well within the near-infrared (NIR) window of 650–1350 nm,^1^ determined by conventional external light excitation at 655 nm (Figure S1) and ^89^Zr-generated CL excitation (Figure 2B).

An initial SCIFI experiment was performed in a multiwell plate format to study the extent of CL-based energy transfer by measuring the total photon flux (photons/second [p/s]) of ^89^Zr and BOD665, both independently and in combination, using an *in vivo* imaging system (IVIS) with external excitation blocked. Wells contained either 1 mM BOD665, 1 MBq of ^89^Zr, or 1 MBq of ^89^Zr in 1 mM BOD665. In isolation, the BOD665 dye generated no fluorescence signal above the background, and ^89^Zr yielded a spectrum representative of its CL energy profile that peaked at 500 nm and then progressively decreased toward higher wavelengths. In contrast, the combination of ^89^Zr and BOD665 yielded a fluorescence spectrum that peaked within the 740 nm emission filter, indicating efficient CL energy transfer to BOD665. A prominent dip in the spectrum between 520 and 680 nm aligns with the BOD665 absorbance band, further supporting this interpretation (Figure 2A,B). These observations are consistent with previous SCIFI studies that describe similar quenching effects.^9^

### Tissue Penetration

**Figure 3:**
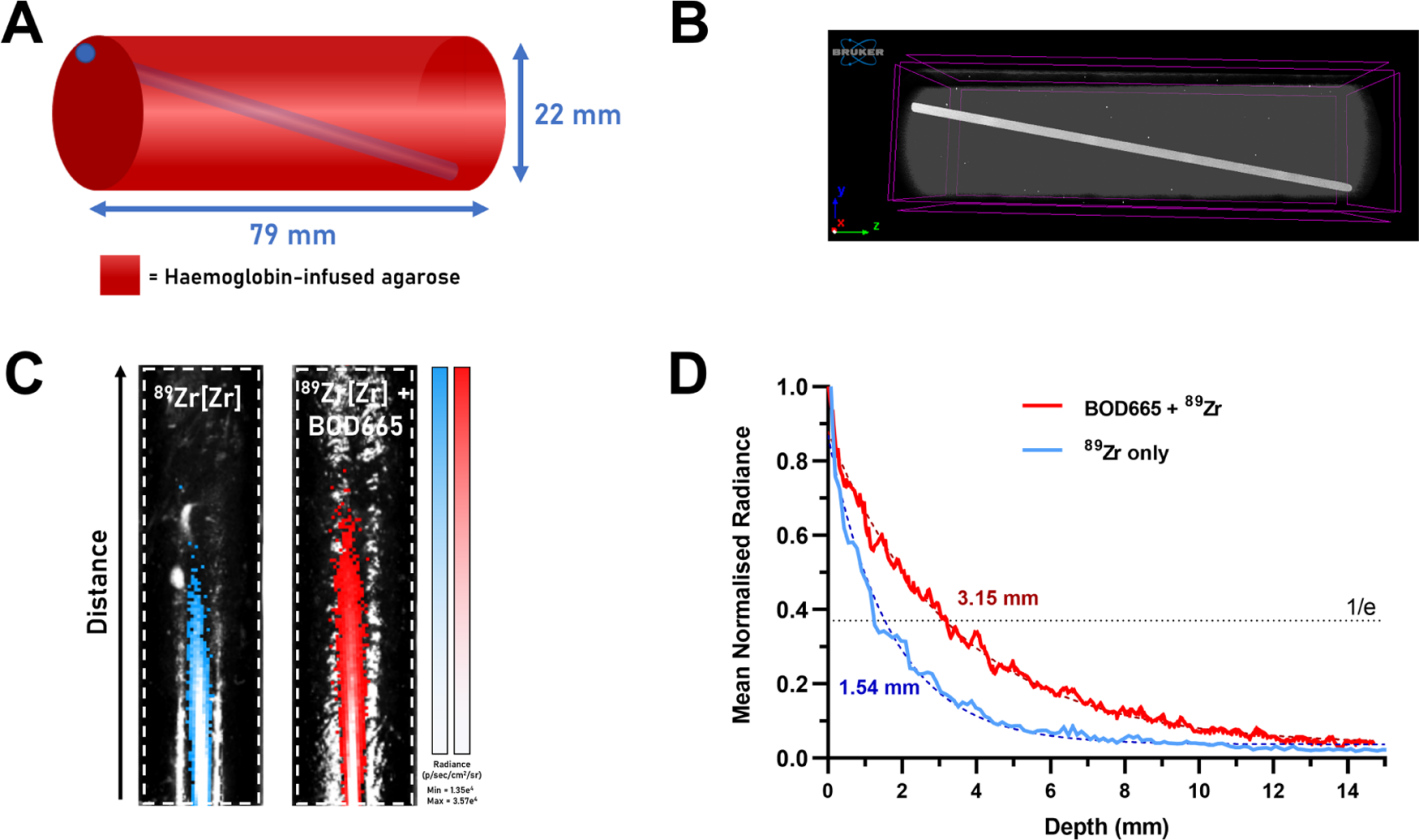
(A) Schematic of hemoglobin-infused agarose optical tissue phantom containing the glass capillary tube (blue). (B) Representative reconstructed CT image of the phantom containing a glass capillary tube filled with 1 mM BOD665 and 1.8–2.8 MBq of ^89^Zr. (C) Representative IVIS images of optical tissue phantoms containing ^89^Zr and ^89^Zr in combination with 1 mM BOD665. (D) Graph of mean-normalized radiance (photons/s/cm^2^/sr) against the depth of the capillary tube in the phantom. 1/e (ca. 37% of incident light) marks the threshold for tissue penetration;^10^ inset values denote the depth (mm) in the phantom that this limit is reached for each condition (*n* = 4).

Penetration depth is defined by the distance through a material at which 1/e (approximately 37%) of the original signal intensity is detectable.^10^ Phantoms loaded with either ^89^Zr and 1 mM BOD665 or ^89^Zr only were imaged on an IVIS and microCT instrument to measure the emission intensity and depth of the emission source, respectively (Figure 3A–D). The addition of BOD665 to ^89^Zr increased the average light penetration depth by 2.05-fold from 1.54 ± 0.04 to 3.15 ± 0.05 mm (Figure 3C,D).

A similar phantom methodology was used by van Oosterom et al. in IVIS-based experiments involving external light excitation of the fluorophore CyAL-5.5_b_ (λ_ex/em_ = 674/693 nm), reporting a ∼ 30% decrease in the fluorescence signal at 7 mm depth and near-background level at 14 mm.^11^ Other studies have adapted capillary tubes to create small “seed” vessels to encapsulate fluorophore solutions. Buckle et al. used dual-emissive quantum dots (em. = 520, 660 nm) in capillary seeds to aid tumor resection and found the 520 and 660 nm emissions to penetrate 5 and 12 mm, respectively, through a porcine tissue phantom following light excitation.^12^ A follow-up study comparing the penetrative ability of fluorophores FITC, TRITC, Cy5.5, and ICG (emissions in 400, 500, 600, and 700 nm ranges, respectively) found that only the latter two dyes were detectable through 14 mm of porcine tissue, due to their longer excitation and emission wavelengths. Combinations of several dyes in multiemissive seeds facilitated the estimation of seed depth within a tissue phantom due to the differences in emission wavelength.^13^ The enhanced penetrability of the fluorophores in these studies compared to the CL excitation of BOD665 can be attributed to the differences in the excitation method, fluorophore spectroscopic properties and concentration, phantom composition, and definition of penetration depth.

### Synthesis and Characterization of DFO-Trastuzumab-BOD665

**Figure 4:**
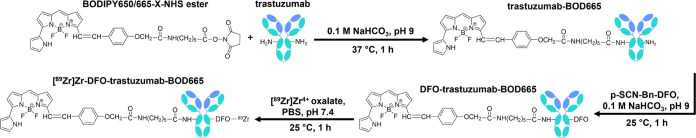
Reaction scheme for the synthesis of the radioimmunoconjugate [^89^Zr]Zr-DFO-trastuzumab-BOD665.

Trastuzumab was modified with BOD650/665-X-NHS ester by stochastic modification of immunoglobulin lysine residues via amide bond formation at ε-amino positions, with yields of 47 ± 16% and degrees of labeling (DOL) of 3.0 ± 0.8, determined using UV–vis measurements. The *A*_max_ of the BOD665 fluorophore following conjugation to the antibody increased from 655 to 665 nm, which is consistent with observations reported for other antibody–fluorophore conjugates.^14^ Next, the bifunctional ^89^Zr chelator p-SCN-Bn-deferoxamine (p-SCN-Bn-DFO) was conjugated to trastuzumab-BOD665 via lysine-directed thiourea formation, generating DFO-trastuzumab-BOD665 in yields of 95.9 ± 5.3% (Figure 4).

**Figure 5:**
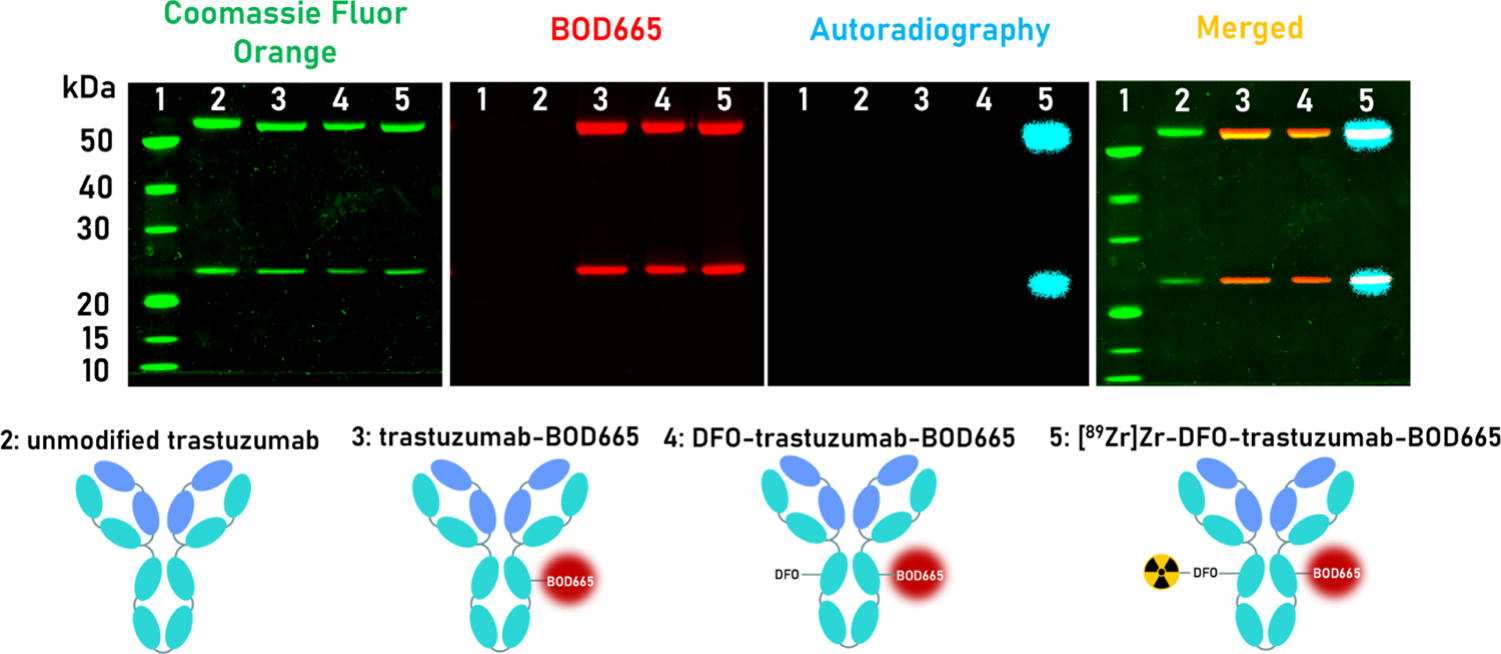
Radio-SDS-PAGE of unmodified trastuzumab (lane 2), trastuzumab-BOD665 (lane 3), DFO-trastuzumab-BOD665 (lane 4), and [^89^Zr]Zr-DFO-trastuzumab-BOD665 compared to a protein ladder. The gel was performed under reducing conditions, stained with Coomassie Fluor Orange, and analyzed through fluorescence and digital autoradiography imaging.

After each synthetic stage, antibody conjugates were analyzed by sodium dodecyl sulfate polyacrylamide gel electrophoresis (SDS-PAGE) under reducing conditions that confirmed the attachment of BOD665 at both the heavy and light chains of trastuzumab (Figure 5).

DFO-trastuzumab-BOD665 was further characterized by matrix-assisted laser desorption ionization time-of-flight (MALDI-TOF) mass spectrometry at each stage of synthesis. By analyzing the shift in mass between unmodified trastuzumab and trastuzumab-BOD665, the DOL_BOD665_ was determined to be 2.64 (Figure S2), which is consistent with the DOL_BOD665_ range established by absorption spectroscopy (3.0 ± 0.8). Furthermore, the DOL_DFO_ was determined to be 1.88 (Figure S2) in good agreement with values reported in the prior literature obtained using the same protocol.^15^

### Radiolabeling and Characterization

DFO-trastuzumab-BOD665 was labeled with neutralized ^89^Zr with a radiolabeling efficiency of 97.1 ± 3.6% and specific activity of 0.1 MBq/μg. Size exclusion chromatography was used to separate [^89^Zr]Zr-DFO-trastuzumab-BOD665 from residual free ^89^Zr, and the immunoSCIFI probe was used in *in vitro* assays when the radiochemical purity (RCP) was ≥95%. [^89^Zr]Zr-DFO-trastuzumab-BOD665 and relevant precursors were further characterized by radio-SDS-PAGE under reducing conditions, and fluorescence imaging and digital autoradiography revealed colocalization of BOD665 fluorescence and ^89^Zr with both the heavy and light chains of trastuzumab in the lane corresponding to [^89^Zr]Zr-DFO-trastuzumab-BOD665 (Figure 5).

**Figure 6:**
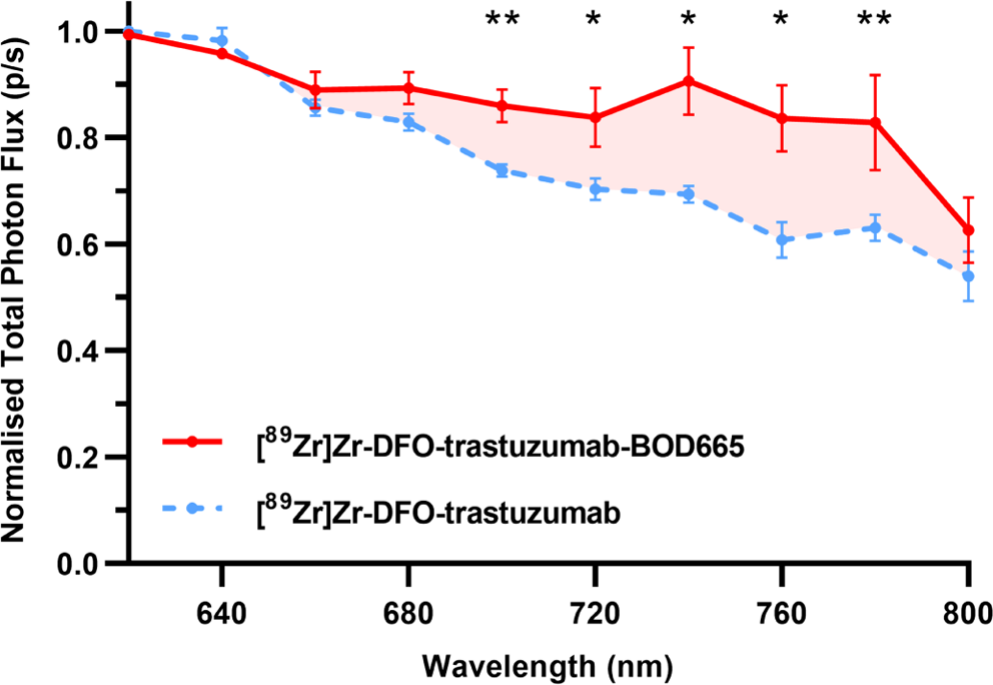
Normalized total photon flux (p/s) measured between 620 and 800 nm from aqueous solutions of [^89^Zr]Zr-DFO-trastuzumab-BOD665 (red) and [^89^Zr]Zr-DFO-trastuzumab (blue) radioimmunoconjugates (*n* = 3).

[^89^Zr]Zr-DFO-trastuzumab-BOD665 was imaged alongside a [^89^Zr]Zr-DFO-trastuzumab control on an IVIS between 620 and 800 nm with excitation blocked. Normalized fluorescence data revealed that the BOD665 radioimmunoconjugate generated higher photon emissions than the control in the region of 660–800 nm (Figure 6), aligning with the BOD665 emission spectrum following incubation with ^89^Zr (Figure 2A).

### Binding Specificity

**Figure 7:**
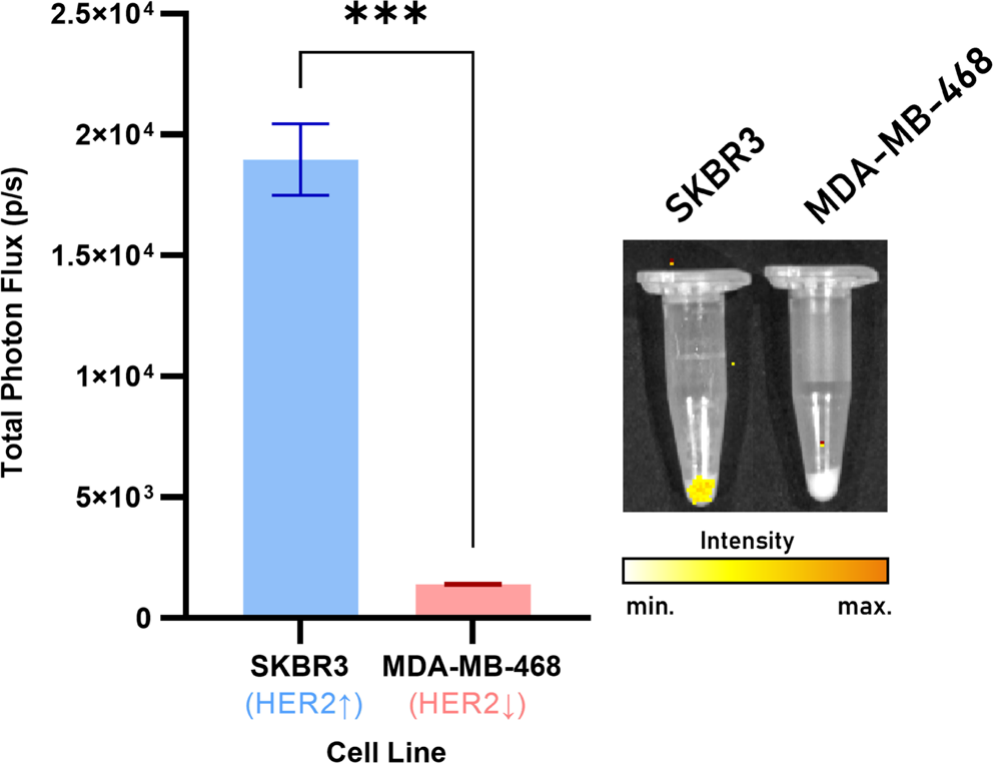
Total photon flux (p/s) measured from SKBR3 (high-HER2) and MDA-MB-468 (low-HER2) cell pellets following incubation with [^89^Zr]Zr-DFO-trastuzumab-BOD665. Representative images of the pellets show molecular targeting of the resulting SCIFI at 740 nm. Data normalized to the cell number in the pellet and to the highest emission value across the pellets. The CL contribution to the emissions at 740 nm has also been subtracted (*n* = 4).

[^89^Zr]Zr-DFO-trastuzumab-BOD665 was incubated with two human breast cancer cell lines, SKBR3 and MDA-MB-468, expressing high and low levels of HER2, respectively.^16^ Cells were condensed into pellets prior to IVIS imaging at 740 nm, with excitation blocked. Analysis of the imaging data demonstrated significantly more immunoSCIFI conjugate bound to the SKBR3 cells compared to the MDA-MB-468 cells (*p* < 0.01), indicating retention of HER2 binding specificity (Figure 7).

## Conclusions

This study describes the synthesis, characterization, and early *in vitro* evaluation of a self-excitatory immunoSCIFI probe that exhibits high binding specificity for the clinically relevant cancer biomarker, HER2, and produces red-shifted photonic emissions with greater depth penetration relative to Cerenkov luminescence. These promising findings suggest a potential role for immunoSCIFI probes in medical imaging applications, e.g. fluorescence-guided surgery.

It is important to acknowledge that the generalizability of these findings is limited by the evaluation of a single immunoSCIFI probe, and the development of this technology will benefit from investigations featuring a broader range of configurations (i.e. antibody, fluorophore, radioisotope combinations) to enhance performance and expand the scope of clinical utility. In addition, clinical translation of this approach will require careful consideration of the radiation dose implications for patients and surgical teams. Lastly, a natural progression of this work is to evaluate immunoSCIFI as a tool for imaging disease biomarkers in an *in vivo* model; an investigation of this nature is currently underway and will be reported in the near future.

## Experimental Procedures

### General Methods

All materials were obtained from Fisher Scientific unless otherwise specified and used without any further purification. Deionized water was obtained using a Select Fusion ultrapure water deionization system (Suez) with a resistance of >18.2 MΩ/cm at 25 °C. Absorbance measurements were obtained using a NanoDrop One^C^ Microvolume UV–vis spectrophotometer (NanoDrop Technologies, Inc.). MALDI-TOF mass spectrometry analysis was performed by using a Bruker Microflex LRF. Radioactivity measurements were obtained by using a CRC-25 dose calibrator (Capintec, Inc.). Radiolabeling of immunoconjugates was verified by instant thin-layer chromatography (iTLC) using glass microfiber chromatography paper (iTLC-SA, Agilent). Autoradiography of radio-iTLC strips was imaged using an Amersham Typhoon bioimager (GE) and analyzed using ImageQuant software (GE Healthcare).

### Conjugation of BOD665 to Trastuzumab

Aliquots of 3 mg of lyophilized trastuzumab were dissolved in 500 μL of 0.1 M NaHCO_3_ (pH 8.3) and washed three times in prerinsed 30 kDa MWCO 0.5 mL centrifugal filters (Amicon) at 12 000*g* for 8 min. Following each spin cycle, 0.1 M NaHCO_3_ (pH 8.3) was added to maintain a total volume of 500 μL. The purified antibody was collected from the filters at 2500*g* for 3 min and the concentration adjusted to 5 mg/mL with buffer based on absorbance measurements. BODIPY 650/665-X NHS ester (1 mg) was dissolved in 50 μL of DMSO to obtain a 20 mg/mL stock solution, aided by sonication for 5 min. From this stock solution, a 50-fold molar excess of the dye was added to trastuzumab (1 mg, 200 μL; DMSO % v/v = 12.5%) and incubated at 25 °C for 1 h at 450 rpm.

### Size Exclusion Chromatography

Crude trastuzumab-BOD665 conjugates were purified using Sephadex-G50 (Sigma-Aldrich) size exclusion chromatography in a 2 mL glass minicolumn, eluting with 100 μL fractions of PBS (pH 7.2). The immunoconjugate eluted between fractions 8 and 12, whereas any unreacted dye remained in the column past fraction 48 when elution was stopped. Fractions were analyzed for absorbance at 280 and 665 nm (absorption maximum for the conjugated BOD665 dye), and those containing the conjugate were combined and concentrated using a single spin cycle in centrifugal filters as previously described.

### Degree of Labeling Determination

The mean number of BOD665 dyes conjugated to each antibody (DOL_BOD665_) in the purified immunoconjugate solution was calculated using the following equations
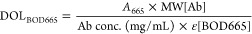
where
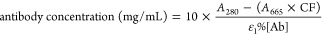
CF = correction factor (*A*_280_/*A*_665_) and ε_1_% = percent molar attenuation coefficient for a 10 mg/mL IgG solution.

The molar attenuation coefficient (ε) of BOD665 was determined from a standard curve via UV–vis absorption spectroscopy for both unconjugated (ε_655_ = 84 590 M^–1^ cm^–1^) and conjugated (ε_665_ = 65 210 M^–1^ cm^–1^) states of the dye.

### DFO Attachment

A 2 mg/mL p-SCN-Bn-DFO (1-(4-isothiocyanatophenyl)-3-[6,17-dihydroxy-7,10,18,21-tetraoxo-27-(N-acetylhydroxylamino)-6,11,17,22-tetraazaheptaeicosine]thiourea) (Macrocyclics) stock solution in DMSO was prepared, and a 10-fold molar excess was added to 100 μL of 5 mg/mL trastuzumab-BOD665 in 0.1 M NaHCO_3_ (pH 8.9–9.0). The reaction mixture was covered to protect it from light and incubated at 37 °C for 1 h at 450 rpm. The resulting DFO-trastuzumab-BOD665 conjugates were purified using centrifugal filters as per the previously described method, and the buffer was exchanged to PBS (pH 7.2).

### MALDI-TOF

The protocol for matrix composition was adapted from Signor et al.^17^ A saturated 40 mg/mL solution of α-cyano-4-hydroxycinnamic acid (α-CHCA) was prepared (solution 1). The matrix solution consisted of a 1:1 ratio of α-CHCA and 2,5-dihydroxybenzoic acid (DHB) precursor solutions. The precursor α-CHCA solution (20 mg/mL) was prepared in 70:30 (% v/v) acetronitrile:5% formic acid. The precursor DHB solution (20 mg/mL) was prepared in 70:30 (% v/v) 70:30 (% v/v) acetronitrile:0.1% trifluoroacetic acid. Equal parts of both precursor solutions were combined to prepare the matrix. All solutions were prepared at room temperature and thoroughly vortexed for ca. 1 min before use. The saturated α-CHCA solution (0.5 μL) was spotted onto a polished steel target plate (Bruker) and allowed to evaporate, leaving a thin layer of α-CHCA. Next, 0.5 μL of ImmunoSCIFI probe sample (10 μM in PBS, pH 7.4) was spotted onto the dried α-CHCA layer, before finally spotting the matrix solution (0.5 μL) into the sample droplet. The spot was allowed to fully dry prior to analysis. ImmunoSCIFI probe samples were reduced into heavy and light chains of the antibody following incubation at 60 °C for 30 min with dithiothreitol (DTT, final concentration 10 mM).

MALDI-TOF mass spectrometry data were acquired using a Bruker Microflex LRF in linear positive mode (laser 60 Hz, ion source 1:19.5 kV, ion source 2:18.15 kV, lens: 7.00 kV, pulsed ion extraction 240 ns, detector gain 2850 V). Data were subsequently processed using Bruker flexAnalysis software (v3.4).

### Radiolabeling

Zirconium-89 in 1 M oxalic acid (PerkinElmer) was neutralized to pH 7 by the addition of 1 M Na_2_CO_3_. pH was measured using narrow-range pH paper 2–3 min after the addition of Na_2_CO_3._ Approximately 10 MBq of neutralized ^89^Zr solution was added to 100 μg of DFO-trastuzumab-BOD665 and DFO-trastuzumab at a specific activity of 0.1 MBq/μg and incubated at 25 °C for 1 h at 450 rpm. Following incubation, the radiolabeling efficiency and radiochemical purity (RCP) of the conjugates were determined by radio-iTLC using 50 mM EDTA as the mobile phase. Radioimmunoconjugates remained on the baseline (*R*_f_ = 0), whereas free ^89^Zr migrated toward the top of the iTLC strip (*R*_f_ = 0.8–1).^18^ RCP of ≥95% was desired for the *in vitro* analysis. [^89^Zr]Zr-DFO-trastuzumab-BOD665 and [^89^Zr]Zr-DFO-trastuzumab controls were made to equal volumes (100 μL) and transferred to a black-sided 96-well plate for IVIS imaging with excitation blocked, emission filters of 620–800 nm (20 nm increments), and exposure time = 5 min. Total photon flux (photons/second; p/s) was measured for each well and normalized to the highest flux to allow for direct comparison.

### Radio-SDS-PAGE

[^89^Zr]Zr-DFO-trastuzumab-BOD665 was analyzed by sodium dodecyl sulfate polyacrylamide gel electrophoresis (SDS-PAGE) under reducing conditions. Antibody samples (≤6.5 μL, concentration-dependent) were prepared by adding 2.5 μL of sample buffer (NuPAGE 4× LDS sample buffer), 1 μL of 500 mM diothiothreitol (DTT), and deionized water (≤6.5 μL, concentration-dependent), to a total volume of 10 μL. The samples were covered with foil and incubated at 70 °C for 10 min at 450 rpm. Protein molecular weight standards (ThermoScientific PageRuler Unstained Broad Range Protein Ladder) and antibody samples were loaded into a 10-well mini-protein gel (NuPAGE 4–12% Bis-Tris, 1.0–1.5 mm) and ran for 50 min at 200 V in NuPAGE 1× MOPS SDS running buffer. The gel was washed three times in 200 mL of deionized water for 5 min prior to staining with 50 mL of Coomassie Fluor Orange protein stain for 1 h. Following staining, the gel was briefly destained by washing for ≤1 min in 1 M acetic acid and then washed once more in deionized water prior to imaging. The gel was scanned using an Amersham Typhoon bioimager (GE) for Coomassie Fluor Orange (Cy2: λ_ex._ = 488 nm, λ_em._ = 525 nm) and BOD665 (Cy5: λ_ex_ = 635 nm, λ_em._ = 670 nm) in fluorescence imaging as well as digital autoradiography imaging.

### Cell Culture

Human breast cancer cell lines SKBR3 (high-HER2) and MDA-MB-468 (low-HER2) were initially obtained from the American Type Culture Collection (ATCC) and stored in liquid nitrogen. The cell lines were retrieved from liquid nitrogen storage and cultured in RPMI 1640 and DMEM media, respectively. Medium was supplemented with 10% fetal bovine serum (FBS, Sigma-Aldrich), 2 mM l-glutamine, penicillin (100 units/mL), and streptomycin (0.1 mg/mL, Sigma-Aldrich). Cells were maintained in a humidified environment at 37 °C with 5% CO_2_ (g) until they reached 80–90% confluency, at which point they were passaged using 0.25% trypsin-EDTA solution (Sigma-Aldrich). Cells were cultured for ≤6 months following retrieval from liquid nitrogen and were tested regularly for the absence of mycoplasma.

### Binding Affinity

SKBR3 and MDA-MB-468 cells were maintained until they reached 85% confluence in the T175 flasks (175 cm^2^ surface area). Accutase solution (Sigma-Aldrich) was added to the cells at 10 mL per 75 cm^2^ surface area, and the flasks were incubated at room temperature for 20 min until the cells detached. Following detachment, the cells were suspended in 15 mL of media and spun down to create a cell pellet (14 000 rpm, 7 min) and remove the accutase. The pellets were resuspended in 10 mL of media, and 65 μg of [^89^Zr]Zr-DFO-trastuzumab-BOD665 (5–6 MBq) was added and incubated for 2 h at room temperature.

Following incubation, the cell suspensions were briefly spun down again to remove the radioactive supernatant from the cells. The resulting pellets were resuspended in 10 mL of PBS (pH 7.2) and washed three times in 3 min spin cycles, removing the supernatant and resuspending each time. Before the final spin cycle, 5 μL of cell suspension was retained for cell counting, using 5 μL of trypan blue to identify viable cells. The final cell pellet was resuspended in minimal PBS, transferred to 1.5 mL LoBind Eppendorf tubes, and spun down (6000 rpm, 5 min) to remove any excess liquid.

The radioactivity of the cell pellets was measured ca. 16 h later, prior to imaging. The pellets were imaged together on an IVIS with excitation blocked, an open emission filter (500–840 nm), exposure time = 5 min. An elliptical region of interest (ROI) was fit around the cell pellet in the tip of the Eppendorf tube, and the total photon flux (photons/second; p/s) was measured for each pellet. Following imaging, the cell pellets were retained for bicinchoninic acid (BCA) protein assays, once radioactivity could no longer be detected, to provide an additional estimate of the number of cells within the pellets. Emission data were normalized to the cell number and to the highest emission value recorded across the pellets. The CL contribution at 740 nm (ca. 28.6%) was also removed, calculated from a previously obtained ^89^Zr-generated CL spectrum (Figure S3).

### Tissue Phantom Preparation

Tissue-simulating optical phantoms were used to evaluate the penetrative ability of ^89^Zr-generated CL and SCIFI resulting from the CL excitation of BOD665. Cylindrical phantoms were created by pouring an agarose–hemoglobin solution into molds created from universal tubes with the conical end removed. Fine glass capillary tubes (0.095 mm internal diameter) were secured at an oblique angle throughout the length of the molds to create a uniform void for the ^89^Zr solutions (Figure 7A). Phantoms were created using a slightly modified version^11^ of an original protocol,^19,20^ where 170 μM lyophilized human hemoglobin (Sigma-Aldrich) was dissolved in a 1% (w/w) agarose (Sigma-Aldrich) solution. The agarose solution was heated in a microwave to dissolve and was allowed to cool to 40 °C before adding the hemoglobin. The mixture was thoroughly combined and then poured into molds and allowed to solidify at room temperature before removal of the phantoms from the molds and careful extraction of the capillary tubes. A narrow vertical slice was cut along the length of the tube on the side opposite to the closed end of the capillary tube to stabilize the phantom for imaging. 1 mM solution of BOD665 with ^89^Zr in DMSO (1.65 ± 0.27 MBq) and ^89^Zr alone in DMSO (2.15 ± 0.52 MBq) solutions were created and used to uniformly fill capillary tubes. The tubes were sealed with epoxy glue and inserted into the agarose–hemoglobin phantoms for imaging.

Phantoms were positioned with the closed end of the capillary tube facing upward on top view and placed into the IVIS. The phantom was imaged with an excitation blocked and an open emission filter (500–840 nm), with an exposure time of 60 s. The radiance (p/s/cm^2^/sr) profile was measured using Living Image (version 4.5.2) software by drawing a line spanning the entire length of the capillary tube. The distance along the tube was converted to the depth of the capillary tube in the agarose–hemoglobin phantom following CT imaging, and the data were fit to a hyperbola model.

The phantoms were positioned in the same orientation as for IVIS imaging and imaged in a Bruker SkyScan 1176 microcomputed tomography (Bruker, Kontich, Belgium) scanner to allow the tube depth within the agarose to be precisely determined. The sample was acquired using the following parameters: source voltage = 45 kV, source current = 556 μA, exposure time = 87 ms, 0.5 mm aluminum filter, 180° scanning with a rotation step of 0.7°, and image pixel size = 35.24 μm. The CT images were reconstructed using NRecon software (Bruker), with a pixel size of 35.278 μm and a smoothing kernel of 2 (Gaussian). 3D-rendered images were visualized using CTVox and processed in DataViewer software (Bruker). The coordinates of the start and end points of the capillary tubes were defined and used to calculate the slope of the capillary tube within the tissue phantom. These data were paired with the distance data obtained from IVIS imaging and used to convert between the two measurements.

### Statistical Analysis

GraphPad Prism v9.4.1 (GraphPad Software, San Diego, CA) was used to create all graphical figures and perform all statistical analysis. A 95% (*p* < 0.05) confidence interval was implemented to determine statistical significance. A two-tailed unpaired *t*-test was performed to compare fluorescence measurements between radioimmunoconjugates (Figure 5) and across two cell lines (Figure 6). All data were obtained at least in triplicate; see figure legends for the sample number (*n*), and results were reported as mean ± standard error of the mean, unless otherwise specified. Statistical significance is indicated by asterisks where ns = not significant, * = *p* < 0.05, and ** = *p* < 0.01.

## Supporting Information Available

Additional spectroscopy data and MALDI-TOF mass spectrometry data (PDF)

## Abbreviations

Ab: antibody
*A*_max_: absorption maximum
BODIPY: boron-dipyrromethene
BOD665: BODIPY 665-X NHS ester
CO_2_: carbon dioxide
DMEM: Dulbecco’s modified Eagle medium
DFO: deferoxamine
DOL: degree of labeling
*E*_max_: emission maximum
[^18^F]FDG: F-fluorodeoxyglucose
IgG: immunoglobulin G
iTLC: instant thin-layer chromatography
CT: computed tomography
IVIS: *in vivo* imaging system
MADLI-TOF: matrix-assisted laser desorption/ionization time-of-flight
MWCO: molecular weight cutoff
NHS: *N*-hydroxysuccinimide
NIR: near-infrared
NIRF: near-infrared fluorescence
PBS: phosphate-buffered saline
PET: positron emission tomography
QD: quantum dot
RCP: radiochemical purity
RIC: radioimmunoconjugate
rpm: revolutions per minute
RPMI: Roswell Park Memorial Institute medium
SDS-PAGE: sodium dodecyl sulfate polyacrylamide gel electrophoresis
^89^Zr: zirconium-89
