# Supplementary material for: Synthesis and In Vitro Evaluation of a HER2-Specific ImmunoSCIFI Probe
Source: ACS Omega. 2023 Dec 7;8(50):47905–12. doi: 10.1021/acsomega.3c06452 (PMC10734019; doi:10.1021/acsomega.3c06452)
Supplement: Supplementary file 1 — ao3c06452_si_001.pdf [file ao3c06452_si_001.pdf]

SUPPLEMENTARY INFORMATION

Synthesis and *in vitro* evaluation of a HER2-specific immunoSCIFI probe

Katie Gristwood[1], Saimir Luli[2], Kenneth S. Rankin[3], James C. Knight[1,*]

[1] School of Natural and Environmental Sciences, Newcastle University, Newcastle upon Tyne, NE1 7RU, UK.

[2] Preclinical In Vivo Imaging, Translational and Clinical Research Institute, Newcastle University, Newcastle upon Tyne, NE2 4HH, UK.

[3] Translational and Clinical Research Institute, Newcastle University, Newcastle upon Tyne, NE1 7RU, UK.

Supplementary Figure S1 – Spectro-fluorophotometer BODIPY emission spectra

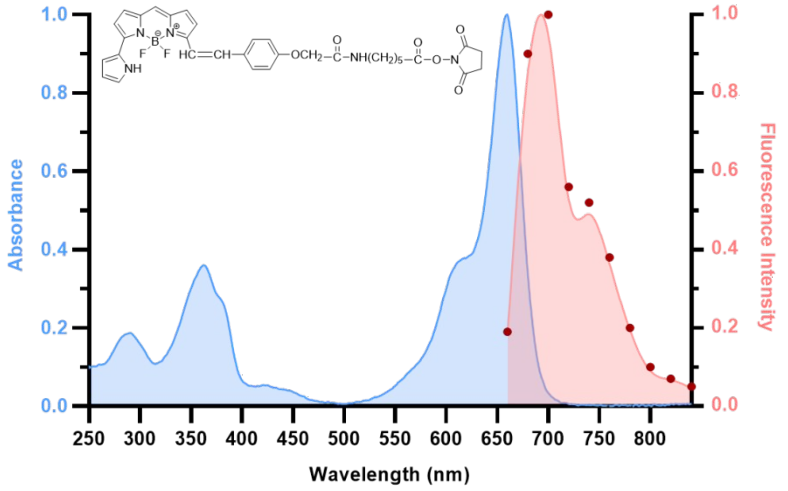

Figure S1. Structure of BOD665 with absorption (blue) and emission (red) spectra in DMSO. Emission spectrum obtained following the addition of 0.25 MBq ^{89}Zr to 1 mM BOD665 solution and imaging between 660-840 nm without external excitation source.

Supplementary Figure 2 – MALDI-TOF analysis

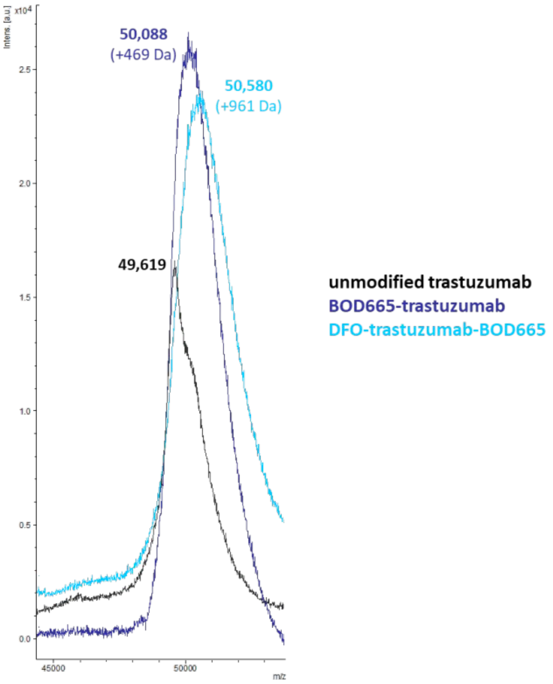

Figure S2. MALDI-TOF analysis of immunoconjugates following addition of BOD665 dye (dark blue), and DFO (cyan). The m/z (Da) for each sample peak corresponds to [M+3]. Expected [m+3] mass shifts resulting from conjugation of BOD665 and DFO units are ca. +178 and +261 Da, respectively.

Supplementary Figure 3 – ^{89}Zr-generated Cerenkov Luminescence spectrum

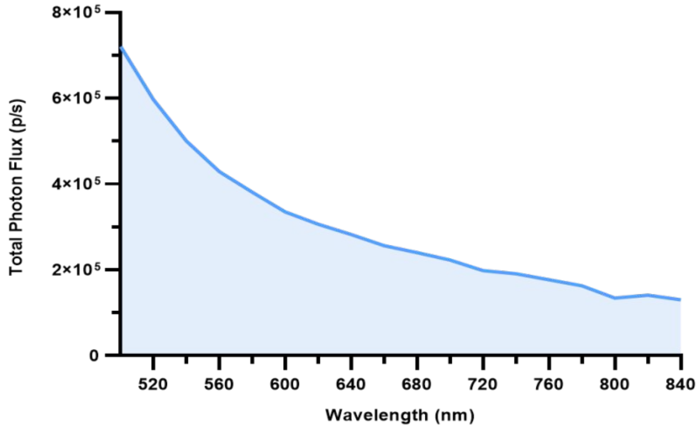

Figure S3. Cerenkov luminescence spectrum of ^{89}Zr-oxalate in PBS. Total photon flux (p/s) was measured on an IVIS spectrum imaging system with emission filters ranging from 500 to 840 nm (20 nm increments). Taken from Gristwood et al. (2022).[1]

References

1. Gristwood, K., Luli, S., Rankin, K. S., Knight, J. C., In situ excitation of BODIPY fluorophores by ^{89}Zr-generated Cerenkov luminescence. *Chem. Commun.* 2022, **58** (83), 11689-11692.
